# Topics of Public Interest

**Published:** 1917-03

**Authors:** 


### TOPICS OF PUBLIC INTEREST

---

**State After-Care of Infantile Paralysis.** Of 2,887 patients reported to the Dept. of Health prior to Oct. 1, 1916, 1,891 had been examined by department surgeons up to Jan. 20. Of the 996 not examined, it is believed that the majority either recovered completely or are under competent orthopaedic care. 801 deaths occurred in the state last year, directly or indirectly from poliomyelitis. 72 clinics have been held since Oct. 17, with an average attendance of 27 patients.

---

**Injunction Against Typhoid Inoculation Refused.** In the quarter-year, July-Sept., 1916, Indianapolis had 600 cases of typhoid and 34 deaths in a population of 295,000. At one time in Aug., the City Hospital had 60 children under treatment at once. Inoculation was made compulsory for school attendance. An application for an injunction by the father of a child, was refused by Judge Lewis B. Ewbank, Sept. 27. His decision was based largely on rulings as to the constitutionality of health boards and statutes requiring small pox vaccination, especially in epidemics.—(Medico-Legal Jour., Oct. 1916).

---

**Compulsory Military Training and Venereal Disease.**. The American Union against Militarism, Washington, has issued an abstract of the testimony of Dr. S. Pollitzer of N. Y. before the sub-committee of the U. S. Senate Committee on Military Affairs, emphasizing the importance of this evil in any system of encampments. Granting this argument to be valid, it must also be granted that a venereal epidemic would also result if the country were invaded on account of unpreparedness. How real this danger is, no one actually knows. But, the same men who would contract venereal diseases in a military encampment of reasonable duration, would, in our estimation, contract them anyhow, even if they do not carry them already. It must be considered also that military discipline allows not only education but arbitrary prophylaxis, to a better degree than civil liberty.

---

**Tuberculosis Decline.** In N. Y. State outside of N. Y. City, there were 5,572 deaths from pulmonary and acute miliary tuberculosis in 1900; 5404 in 1915, the population having meantime increased from 3,836,038 to 4,640,523, so that the rate per 1000 population declined from 1.453 to 1.165. Or, to put the same fact in different terms, a little less than 82% died, as compared with the expectation on the same degree of prevalence as in 1900. The decline has been progressive, with just enough fluctuation from year to year to impress one with its genuineness. We venture to repeat a prophecy made a few years ago, that just as soon as the decline of this disease becomes sufficient so that it is no longer a question of how many chances a month, every one has of suffering an implanting of bacilli which may or may not develop according to his resistance, but of whether the average individual does or not come in contact with bacilli, say once a month or once a year, the decline will be much more rapid and will follow approximately a downward geometric series instead of an arithmetic series.

---

**The Bubbling Fountain** is stated by the Ind. State Med. Assn.' Jan., to have been the source of an epidemic of streptococcus amygdalitis at the University of Wis. Streptococci were found on more than half of the fountains, lodged on the metal piece surrounding the mouth of the water pipe. (Note.

We have often wondered just how effective the current of water might be against the rinsing of various mouths, including sometimes those of dogs, birds and horses and against the grimy hands of boys who squirt water from these sanitary (?) supplies. Ed.)

---

**German Wounded.** Of those wounded in the second year of the war, 70% recovered sufficiently to return to the trenches, 6.4% were permanently disabled, the remainder being fit for home service. Epidemic diseases dropped from 51: 1000 for the first year to 38: 1000 for the second year. 21.5: 1000 were nervous, due to the strain of warfare. Pleurisy amounted to 6: 1000 and pneumonia to 4; 1000. Small pox has entirely disappeared and typhus, typhoid, cholera, etc., have been practically eliminated. The total morbidity has dropped from 120 to 100: 1000, according to monthly reports. (Note: If the 1000 basis is, for all these figures, the total number of troops, it will realize that there is a narrow margin for trivial ailments and that there is an average diablement from disease alone, of about 7%, all the time, beside temporary disablement from disease, wounds and death. Ed.)

---

**Drugless Healers.** The California State Jour. of Med., Feb., speaks favorably of the California law which divides all practitioners into two classes: Physicians and surgeons, who are authorized "to use drugs...... and any or all other methods in the treatment of diseases, injuries, deformities or other physical or mental conditions" and "Drugless Practitioners, who are authorized to practice" without the use of drugs and without in any manner severing or penetrating any of the tissues of human beings except the severing of the umbilical cord." Reasonable educational requirements are demanded for either class of license and diplomas must be presented from schools approved by the Board, and certificates of good moral character. We noted in our Feb. issue that the law had been declared constitutional by the U. S. Supreme Court, in dismissing suits brought by a chiropractor and a "drugless ophthalmologist." We understand that Christian Scientists and others claiming to heal by prayer are exempt. We confess also that, somehow, we had understood that the law was regarded unfavorably by the medical profession. The California State Journal, though admitting that the law has some defects, regards it as a strong protection and warns that it must not be weakened, especially emphasizing the value of the control of the Board which has compelled certain institutions to make radical improvements or go out of business. The division of all practitioners into two classes seems simple and the definition of the respective powers of medical licentiates and what the medical profession rather scornfully likes to call "id omne genus" seems also to have been reduced to remarkably simple and clear language although it occurs to us that, by a quibble, barbers and manicurists might be indicted as severing human tissues. It is worth while for the medical profession of other states to study this law and its practical results, carefully.

---

**A New Medical Research Work.** T .D. Crothers, M. D., Hartford, Conn. A new Research Foundation has been organized at Hartford, Conn., to inquire into the causes and conditions both physical and psychical which antedate all use of spirits. For a long time there has been a growing conviction founded on observation and exact studies, that alcoholism and inebriety, were symptoms of conditions that had been developed long before. It has also been stated that when these conditions were known and properly treated, future spirit and drug taking would be practically stamped out. Wherever the phenomena of the drink evil has been studied, most perplexing symptomology and paradoxical progress has been noted. If the opinion of laymen was correct and alcohol was the only and specific cause, there would be certain uniformity of growth and development and the removal of the cause would be followed by complete restoration. This, does not follow, showing that other causes are influential beyond that of alcohol. What these are is practically unknown. The literature of opinions and theories is very extensive but the range of verifiable facts are small and outside of the laboratory work, are largely unknown. Studies of preventive medicine show that the most important field is to find the actual causes and remove them. Most startling examples are evident in the tracing of the causes of fevers back to their sources and removing these. In this way, several great scourges have been practically driven out. These studies show that the armies of drink and drug takers of today have been literally, born, bred and developed by conditions in the past, also, that the armies of drinking men in the future are growing up now in our own surroundings—not in the slum districts but in the homes of all grades of society. If we could know the causes and conditions, that produce these defectives a new field of medical practice would be opened. Thoughtful physicians everywhere recognize the range of cause and effect and the region of physical and psychical laws that will produce drink and drug takers and other neurosis in the future with absolute certainty. There is a great wealth of facts here which have never been studied and this research foundation proposes to occupy this new field. What is called Psycho-Analysis in the largest meaning of this word, has never been put to service, in the study of these neurotics. Here there is a great wealth of facts and it is assumed that most astonishing results will follow, when these facts are correlated and put to service in actual practice. There are many problems in the drink question that are explained by theories that are unverifiable. The laboratories have shown that alcohol is a narcotic and depressant and this discovery has practically changed medical practice respecting alcohol as remedy. Equally startling discoveries await exact studies in this field of causes back of the use of alcohol, evidently, public sentiment is rapidly forcing the alcoholic question into prominence and every community will be called on to express opinions and to take an active part in the measures for relief. This is literally a physician's problem and to them will be referred the disputed questions. This new work proposes to furnish facts and data from which, they can form conclusions and in this respect, is the last great scientific effort, to point out causes and means and methods for removal above sentiment and theory. Its practical character and the possibilities from such a study appeal to everyone both laymen and physicians as an effort requiring the warmest endorsement and support.

---

**Centenarian.** Mrs. Samuel Sipe, aged 104, a friend of Molly Pitcher, died at Carlisle, Pa., Feb. 10. She was born in Switzerland but had lived in Carlisle over a century.

---

**Rabies.** Dr. W. A. Scott, Health Officer of N. Tonawanda reports that 10 dogs died of that disease in Jan. 105 dogs were taken to the pound and 76 were killed. 3 human beings were bitten by dogs.

---

**Flies.** Remember that the winter and spring are the seasons at which the killing of individual flies is worth while. Prepare early for screening and the use of traps, and see that accumulations of garbage and manure are disposed of before warm weather sets in.

---

**McIntire Prize.** This is established by the American Academy of Medicine, in honor of the completion of 25 years of service as Secretary, of Dr. Charles McIntire of Easton, Pa., one of its founders in 1876. It will be awarded triennially, the amount not being as yet definitely stated. The subject for competition in 1918 is "The Principles of Governing the Physician's Compensation in the Various Forms of Social Insurance.'' The competition open to all. Essays must be type-written, in English, between 5,000 and 20,000 words in length, exclusive of tables, must be submitted under the usual conditions of secrecy, on or before Jan. 1, 1918. The chairman of the committee of awards is Dr. John L. Heffron, Syracuse.

**Alexander Graham Bell Grosvenor Memorial Prize.** The Volta Bureau report that the contest closed last fall resulted in no award (possibly because the essays were very narrowly limited to 20,000-21,000 words). The contest will, therefore, be open to essays received before Nov. 1, 1917. The subject is the home training of deaf children and the prize is $300. Those who contemplate competing should secure full details from the Supt. of the Volta Bureau, 1601 35th St., N. W., Washington. This Bureau conducts an excellent journal, dealing with the instruction of the deaf and the study of phonetics and will send, on application, valuable literature, to any deaf person or anyone interested in the care of the deaf. It does not give medical advice, has no drugs nor instruments for sale, and conducts no school, so that it has the cordial support of the medical profession.

**Civil Service Positions.** The following may interest medical men and women. For most positions, U. S. citizenship, 3 month's residence in N.Y. State and at least 21 years in the world, are required. Apply for forms to the Civil Service Commission, Albany, not later than Mch. 19. Examinations will be held in several places, to be determined later, on March 31.

78. Assistant Chemist, Public Service Commission, First District. $1201 to $1500. Men only. Candidates must be graduate chemists with at least two years' practical experience. The examination will presuppose a familiarity with analyses of steel and cast iron, cement, asphalt, coal tar pitches, mixed paints, dry pigments, etc., water analyses, gas-testing methods, use of the microscope and general chemistry. Subjects of examination and relative weights: Written examination, 6; education and experience, 4. Open to non-residents. No sample questions.

79. Water Analyst, State Department of Health. $1600. Men only. Appointees must have a thorough training in analytical chemistry, with some practical laboratory experience in water analysis. Subjects of examination and relative weights: General and sanitary chemistry and water analysis,

6; education and experience, 4. Open to non-residents and non-citizens. No sample questions.

80. Laboratory Assistant in Bacteriology, State Department of Health. Men and Women. $720 to $1200. Open to non-residents and non-citizens. Only candidates who have satisfactorily completed a systematic course in bacteriology and have also had not less than three months' practical experience in laboratory work will be accepted. Candidates will be examined in the technical procedures used in the study of pathogenic bacteria of infectious disease, and immunity, and the standard methods used in the examination of milk, water, sewage, air and soil. Subjects of examination and relative weights: Written examination relating to the duties of the position, 6; education and experience, 4. No sample questions.

81. Director of the Division of Vital Statistics, State Department of Health. Salary $4000 a year. Open to men only. Candidates must have had not less than five years' experience in the collection, interpretation and publication of vital statistics. The Director of the Division of Vital Statistics is the administrative and statistical head and has full charge of the work of the Division. He supervises the collection of certificates of births, deaths and marriages occurring in the State which are filed with the Department, and outlines and supervises the tabulation of compilation of the statistical items on the certificates, and analyzes same for monthly and annual reports of the Department. He aids in enforcing the provisions of the Vital Statistics Law requiring the complete registration of all births, deaths and marriages occurring in the State, makes special investigations as to the completeness of the registration, and conducts such special statistical investigations as may be required by the State Commissioner of Health. Subjects of examination and relative weights: Written examination relating to the duties of the position, 2; oral examination, 4; experience and personal qualifications, 4. Open to non-residents. No sample questions.

82. Prison Physician, State Prisons and Reformatories. Salary, $2000 without maintenance. Examination open only to men who are licensed medical practitioners in this State. It is desired to secure applicants who since graduation have had at least one years' experience on the resident medical staff of a general hospital. Subjects of examination and relative weights: Written examination pertaining to the duties of the position, 6; education and personal qualifications, 4. Candidates who attain at least 60 per cent. in the written examination may be summoned before the examining committee in connection with the rating of the last mentioned subject. It is expected that one appointment will be made at Sing Sing prison in the near future, where the appointee will be fully responsible for the medical service, including hospital.

83. Assistant Prison Physician, State Prisons and Reformatories. Salary, $1500 without maintenance. Examination open only to men who are licensed medical practitioners in this State, and preference will be given to those who since graduation have had at least one year's experience on the resident medical staff of a general hospital. Subjects of examination and relative weights: Written examination, pertaining to the duties of the position, including laboratory work, prison sanitation and hygiene, 6; education, experience and personal qualifications, 4. Candidates who attain at least 60 per cent. in the written examination may be summoned before the examining committee in connection with the rating of the last mentioned subject. It is expected that one appointment will be made in the near future at Sing Sing prison.

84. Assistant Physician, Regular or Homeopathic. This examination is intended to provide eligibles for the position of assistant physician in the State hospitals and for other positions of a similar nature in various State and County institutions. Salary in the State hospitals $1200, increasing $100 each year to $1600, with maintenance, including quarters, board, laundry, etc. Examination open to men and women who are licensed medical practitioners in this State, who have graduated from a registered medical school and who since graduation have had one year's experience on the resident medical staff of a general hospital, or as medical interne or clinical assistant in a State hospital or institution or have been engaged for three consecutive years in the practice of medicine. Subjects of examination and relative weights: Written examination, covering anatomy, physiology, chemistry, materia medica, therapeutics, obstetrics, surgery, theory and practice, 7; education, experience and personal qualifications, 3. Candidates who attain at least 60 per cent. in the written examination may be summoned before the medical examining committee in connection with the rating of the last mentioned subject. Open to non-residents. Unmarried men preferred.

---

**Swindler Arrested.** Feb. 16, a man was arrested in Buffalo who is alleged to have swindled a number of physicians by delivering a package containing an empty bottle, stated to have been ordered from some supply house. His calls were timed so as to reach the office in the doctor's absence. We wonder if the C. O. D. method was successful in the case of some of our old subscribers who have not paid their subscription bills for several years.

---

**Examination for the Position of Assistant Surgeon in the U. S. Public Health Service** will be held monthly at 8 cities, including N. Y. Application should be made to the Surgeon General at Washington, to secure an invitation. Applicants must be between 23 and 32 years old.

---

**Anthrax.** Several cattle and horses were found to be suffering from anthrax on a farm near Corning, Jan. 30. Infection is supposed to have been due to drinking water from the Tioga River, contaminated by tanneries up-stream in Pennsylvania.

